# Improving Physician Orders for Life-Sustaining Treatment (POLST) Documentation Through an Age-Friendly Health Systems 4Ms What Matters Workflow in an Outpatient Geriatric Clinic

**DOI:** 10.7759/cureus.119085

**Published:** 2026-11-03

**Authors:** Kourosh A Moshiri, Louis Guitron, Hyo Mun

**Affiliations:** 1 Geriatrics/Internal Medicine, Eisenhower Medical Center, Rancho Mirage, USA; 2 Geriatrics, Eisenhower Medical Center, Rancho Mirage, USA

**Keywords:** 4ms, advance care planning, age-friendly health systems, geriatrics, polst, quality improvement

## Abstract

Background: Physician Orders for Life-Sustaining Treatment (POLST) translate treatment preferences into portable medical orders for people with serious illness or advanced frailty. We evaluated POLST documentation after integrating an advance care planning prompt into an Age-Friendly Health Systems 4Ms What Matters workflow in an outpatient geriatric clinic.

Methods: This single-center quality improvement project compared aggregate provider data for 2024 and 2025 in a clinic serving approximately 2,000 adults aged 65 years or older. Beginning in January 2025, a standardized electronic health record template incorporated the 4Ms and POLST prompts. Implementation included individual provider training, staff instructions, and quarterly one-to-one provider feedback on all 4Ms measures with encouragement to use the templates. Reported POLST documentation proportions used counts of encounters associated with scanned forms and total encounter denominators. Analyses were descriptive; monthly Provider C percentages were plotted without statistical process control limits.

Results: Overall documentation increased from 11.7% (335/2,865) to 26.9% (1,158/4,305), an increase of 15.2 percentage points and approximately 130% relative to baseline. Provider-level proportions increased from 4.8% to 15.9% for Provider A, 7.3% to 10.1% for Provider B, and 21.7% to 61.9% for Provider C. Provider C reached at least 84.5% in each of the final four months of 2025. The investigator's account of his own workflow identified in-visit discussion and form completion as practices warranting further study.

Conclusions: An Age-Friendly Health Systems 4Ms What Matters workflow was associated with increased reported encounter-level POLST documentation, with substantial variation among clinicians. The findings support further testing of conversation training and workflow support. This uncontrolled encounter-level evaluation does not establish causality, new form completion, or goal-concordant care.

## Introduction

Physician Orders for Life-Sustaining Treatment (POLST) translate discussions about treatment preferences into portable medical orders. POLST is intended primarily for people with serious illness or advanced frailty, and completion is voluntary. Advance care planning (ACP) is appropriate more broadly, whereas a POLST completion target requires attention to clinical appropriateness and patient choice [1].

The evidence linking POLST to subsequent care is largely observational. A systematic review of 20 studies involving 104,554 patients found moderate-strength evidence that treatment-limiting POLST orders may reduce treatment intensity near the end of life [2]. An integrative review found associations between POLST use and several quality-of-care outcomes, including concordance between treatment and documented orders, but emphasized the need for stronger prospective evidence [3]. Among patients hospitalized near the end of life, treatment-limiting POLST orders were associated with lower intensive care unit admission rates; however, some patients still received care that was potentially discordant with their orders [4]. These findings support distinguishing documentation from the quality of the conversation and the care subsequently delivered.

Age-Friendly Health Systems organize care for older adults around What Matters, Medication, Mentation, and Mobility. The What Matters domain provides a framework for eliciting patients' goals and aligning care with their preferences [5]. Electronic health record (EHR) measures can support implementation, although documentation measures require careful definition and validation [6,7]. ACP improvement initiatives in primary care have used learning collaboratives, staff facilitation, workflow redesign, and performance feedback, with variable results [8,9].

At our outpatient geriatric clinic, baseline POLST documentation was approximately 12% and varied among clinicians. Using the Model for Improvement, the project's stated aim was to at least double the proportion of encounters associated with a POLST form documented in Epic among patients aged 65 years or older within 12 months by incorporating a POLST prompt into the What Matters component of a standardized 4Ms visit template. We report annual provider-level documentation results. An exploratory description of the investigator's clinical workflow was used to identify candidate changes for the next improvement cycle.

## Materials and methods

Study design and setting

This single-center, uncontrolled before-and-after quality improvement project was conducted in the outpatient geriatric clinic at Eisenhower Health in Rancho Mirage, California. The clinic served a panel of approximately 2,000 adults aged 65 years or older and was staffed by three physicians, reported as Providers A, B, and C. The baseline period was January through December 2024, and the intervention period was January through December 2025. The standardized template was introduced in January 2025.

Clinical considerations for offering POLST to adults aged 65 years or older included serious illness, advanced frailty, multiple comorbidities, or an estimated life expectancy of approximately one year or less, assessed through clinician judgment and individualized shared decision-making. Patients aged 65 years or older who wished to complete a POLST were also considered, without requiring a life expectancy under one year; this local approach was informed by Bomba and colleagues' description of interested people of advanced age, without a specified age cutoff [10]. The reporting denominators included all recorded provider encounters for patients aged 65 years or older in both years, without exclusions based on these clinical considerations. POLST completion remained voluntary and guided by clinical appropriateness and patient preferences.

Intervention and improvement approach

During 2024, providers were encouraged to address POLST with their patients. The initial change concept for 2025 was a standardized EHR visit template incorporating all four Ms and a dedicated What Matters section. The establish-care template asked "(POLST) addressed this visit", and the follow-up template asked "(POLST) in chart". The proposed mechanism was that a visible prompt linked to goals-of-care discussion would make the workflow more reliable. Template responses alone did not define the scanned-form outcome.

The Plan-Do-Study-Act approach comprised planning the documentation prompt, implementing the template in January 2025, studying provider-level performance, and identifying changes for the next cycle. In 2025, the principal investigator trained each provider individually on the Age-Friendly template, including POLST. During visits, clinicians used the template prompt to consider whether a POLST discussion was appropriate. Patients who met the clinical considerations above, or who expressed interest, were offered the opportunity to complete a POLST. The principal investigator, with the clinic administrator, instructed staff to provide the California POLST form to willing patients on the encounter date. Every three months, the principal investigator provided one-to-one feedback on all 4Ms measures and encouraged template use. The planned second phase would test a more standardized POLST discussion and completion workflow, with conversation guidance and practical support during visits.

Measures and data collection

The primary process measure was the reported monthly proportion of provider encounters associated with a scanned POLST in Epic Media. The numerator counted encounters with a newly completed or previously scanned POLST; the denominator counted all encounters for patients aged 65 years or older. Counted forms had been completed by the encounter date; new forms were completed during visits and scanned that day or afterward. A template entry describing discussion, review, deferral, or clinical inappropriateness without a scanned POLST form was not sufficient to meet this document-presence measure. An information technology (IT) representative generated monthly provider-level reports using Epic searches for POLST forms in Media. The export identified month (Calendar_Yr_Month), provider (Visit_Provider), the POLST documentation count (Polst_Total), and the encounter count (Total_Patients). Despite its label, Total_Patients represented encounters in this project. The IT team and the principal investigator manually verified all reported POLST-positive encounters against the scanned forms in Media. IT used the same counting and identification methods in both years. Monthly counts were summed to obtain the annual numerators and denominators. Monthly source counts are reported in the Results section.

The unit of analysis was the encounter. Each distinct encounter contributed at most one positive count, even if more than one POLST form was present. No patient-level deduplication or longitudinal linkage was performed in this aggregate analysis. A patient could contribute through repeated visits, including visits associated with an existing form. Consequently, the measure reflects reported encounter-level documentation and should not be interpreted as the number of unique patients, newly completed forms, or encounters in which a POLST conversation or form review occurred. New forms and previously completed forms were not separately quantified.

Clinic-level proportions were calculated by summing provider numerators and denominators before division, rather than averaging provider percentages. Provider-level proportions and absolute changes in percentage points were also calculated. Monthly percentages for Provider C were examined to describe the pattern during implementation. We did not use a universal POLST documentation benchmark.

Analysis

Analyses were descriptive. The absolute change was the 2025 proportion minus the 2024 proportion; the relative change was this difference divided by the 2024 proportion. To examine the influence of changing provider encounter shares, an exploratory calculation applied the 2024 provider encounter weights to the 2025 provider-specific documentation proportions. This standardization addressed provider weights only and did not adjust for patient characteristics.

No patient-level adjustment for case mix, repeated encounters, or clustering was performed. Monthly Provider C percentages were displayed as a descriptive time series without control limits. We did not classify special-cause variation or infer a statistically confirmed process shift.

Workflow description

Provider C was the corresponding author and principal investigator. The account of his clinical workflow was used to illustrate practices that could inform the next improvement cycle. It is presented as a description of the investigator's own practice rather than an independent assessment of clinician behavior or a formal qualitative study. No standardized observation counts, observation schedule, or comparative workflow assessment of Providers A and B are reported. This description was used to generate hypotheses and did not establish that a particular behavior caused improvement.

The report was organized using the Standards for Quality Improvement Reporting Excellence (SQUIRE 2.0) framework [11].

## Results

Overall documentation

The reported encounter denominator increased from 2,865 in 2024 to 4,305 in 2025, a 50.3% increase. The documentation numerator increased from 335 to 1,158. Overall documentation rose from 11.7% to 26.9%, an absolute increase of 15.2 percentage points and a relative increase of approximately 130%. This exceeded the project's stated doubling target. Annual results by provider are shown in Table 1. Provider B's lower encounter volume early in 2024 reflected his geriatric fellowship training. In 2025, Provider B had more clinical hours and patient appointment slots, while Providers A and C had allocated administrative time. These differences in clinical availability contributed to the changing encounter volume and provider mix. Table 2 shows the monthly source counts.

**Table 1:** Annual POLST documentation by provider Rates are calculated from the reported counts. Overall rates use summed numerators and denominators. Repeated visits by a patient may contribute to these encounter counts. They do not establish unique patients or newly completed forms. n: reported encounter count associated with a scanned POLST form; N: total recorded encounter count for patients aged 65 years or older; POLST: Physician Orders for Life-Sustaining Treatment

Provider	2024 n/N (%)	2025 n/N (%)	Absolute change percentage points
Provider A	62/1,282 (4.8%)	201/1,266 (15.9%)	+11.0
Provider B	36/491 (7.3%)	180/1,784 (10.1%)	+2.8
Provider C	237/1,092 (21.7%)	777/1,255 (61.9%)	+40.2
Overall	335/2,865 (11.7%)	1,158/4,305 (26.9%)	+15.2

**Table 2:** Monthly POLST documentation and provider encounter counts, 2024-2025 Percentages are calculated from the displayed counts and rounded to one decimal place. These are encounter-based counts, not unique-patient counts. Provider B had no visits in July 2024 while on PTO; the IT export contained no row for that month, which is displayed as 0/0 (N/A). Annual totals reproduce Table 1. The source is the POLST Documentation worksheet in the project performance workbook. POLST: Physician Orders for Life-Sustaining Treatment; n: encounters with a documented POLST; N: total provider encounters; N/A: not applicable because no encounters occurred; PTO: paid time off

Month	Provider A n/N (%)	Provider B n/N (%)	Provider C n/N (%)
Jan 2024	3/119 (2.5%)	4/13 (30.8%)	14/61 (23%)
Feb 2024	6/104 (5.8%)	2/8 (25%)	16/75 (21.3%)
Mar 2024	5/133 (3.8%)	4/15 (26.7%)	23/103 (22.3%)
Apr 2024	5/146 (3.4%)	1/3 (33.3%)	25/112 (22.3%)
May 2024	6/113 (5.3%)	7/19 (36.8%)	16/92 (17.4%)
Jun 2024	3/85 (3.5%)	2/16 (12.5%)	23/90 (25.6%)
Jul 2024	6/107 (5.6%)	0/0 (N/A)	18/78 (23.1%)
Aug 2024	4/70 (5.7%)	3/56 (5.4%)	14/69 (20.3%)
Sep 2024	8/94 (8.5%)	5/53 (9.4%)	14/84 (16.7%)
Oct 2024	7/133 (5.3%)	3/73 (4.1%)	19/112 (17%)
Nov 2024	7/129 (5.4%)	2/98 (2%)	31/122 (25.4%)
Dec 2024	2/49 (4.1%)	3/137 (2.2%)	24/94 (25.5%)
2024 total	62/1,282 (4.8%)	36/491 (7.3%)	237/1,092 (21.7%)
Jan 2025	8/137 (5.8%)	4/139 (2.9%)	28/120 (23.3%)
Feb 2025	8/114 (7%)	3/99 (3%)	30/91 (33%)
Mar 2025	5/109 (4.6%)	9/155 (5.8%)	35/103 (34%)
Apr 2025	10/107 (9.3%)	17/177 (9.6%)	55/129 (42.6%)
May 2025	18/111 (16.2%)	16/139 (11.5%)	57/99 (57.6%)
Jun 2025	12/90 (13.3%)	19/162 (11.7%)	65/104 (62.5%)
Jul 2025	19/109 (17.4%)	20/140 (14.3%)	61/79 (77.2%)
Aug 2025	15/79 (19%)	15/146 (10.3%)	88/113 (77.9%)
Sep 2025	21/118 (17.8%)	19/158 (12%)	82/97 (84.5%)
Oct 2025	33/112 (29.5%)	15/154 (9.7%)	99/112 (88.4%)
Nov 2025	22/81 (27.2%)	20/156 (12.8%)	87/103 (84.5%)
Dec 2025	30/99 (30.3%)	23/159 (14.5%)	90/105 (85.7%)
2025 total	201/1,266 (15.9%)	180/1,784 (10.1%)	777/1,255 (61.9%)

Provider variation and monthly pattern

Documentation increased for all three providers (Table 1). Provider C had the largest gain (+40.2 percentage points), followed by Provider A (+11.0) and Provider B (+2.8).

Provider B's encounter share increased from 17.1% to 41.4%, while Provider C's fell from 38.1% to 29.2%. Holding 2024 provider weights fixed yielded a 2025 documentation proportion of 32.4%, compared with 26.9% using the observed 2025 provider mix.

Provider C's monthly documentation rose from 23.3% in January 2025 to 85.7% in December, peaking at 88.4% in October; the final four months all reached at least 84.5%. The 2024 monthly rates ranged from 16.7% to 25.6%. Figure 1 shows the annual documentation comparisons by provider, and Figure 2 shows Provider C run chart time series. Table 2 provides the counts underlying these monthly percentages.

**Figure 1:**
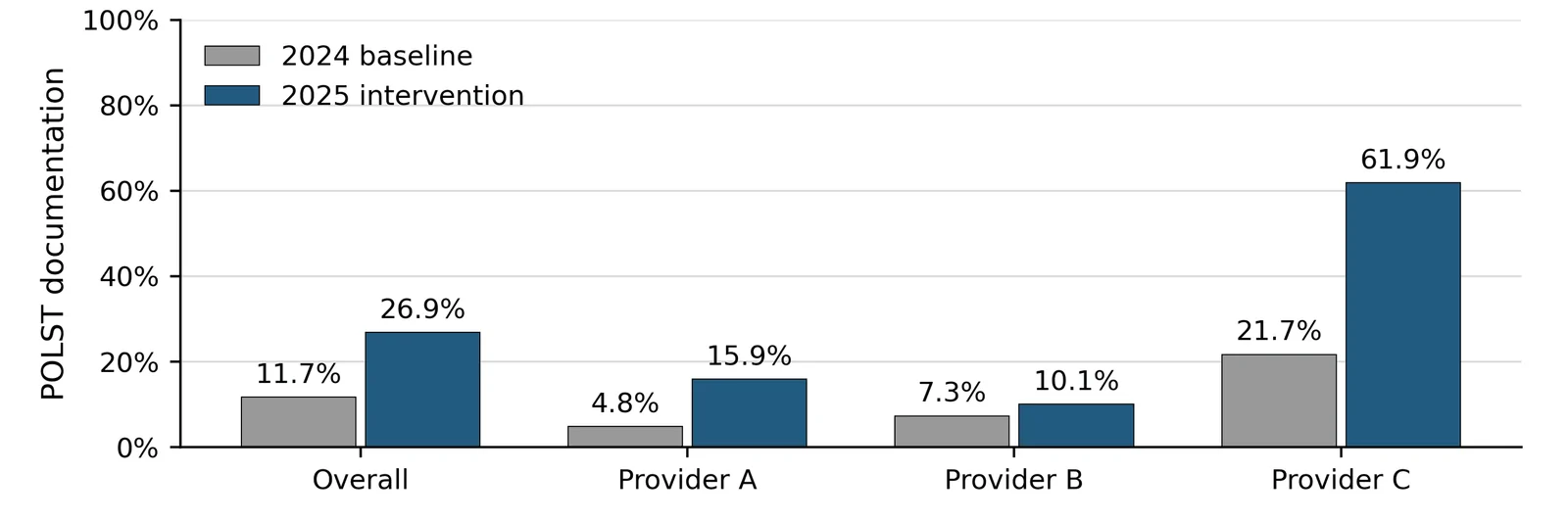
Annual POLST documentation by provider and overall POLST documentation before and after implementation of the What Matters workflow. Annual proportions for the clinic overall and each provider, calculated from the counts in Table 1. POLST: Physician Orders for Life-Sustaining Treatment

**Figure 2:**
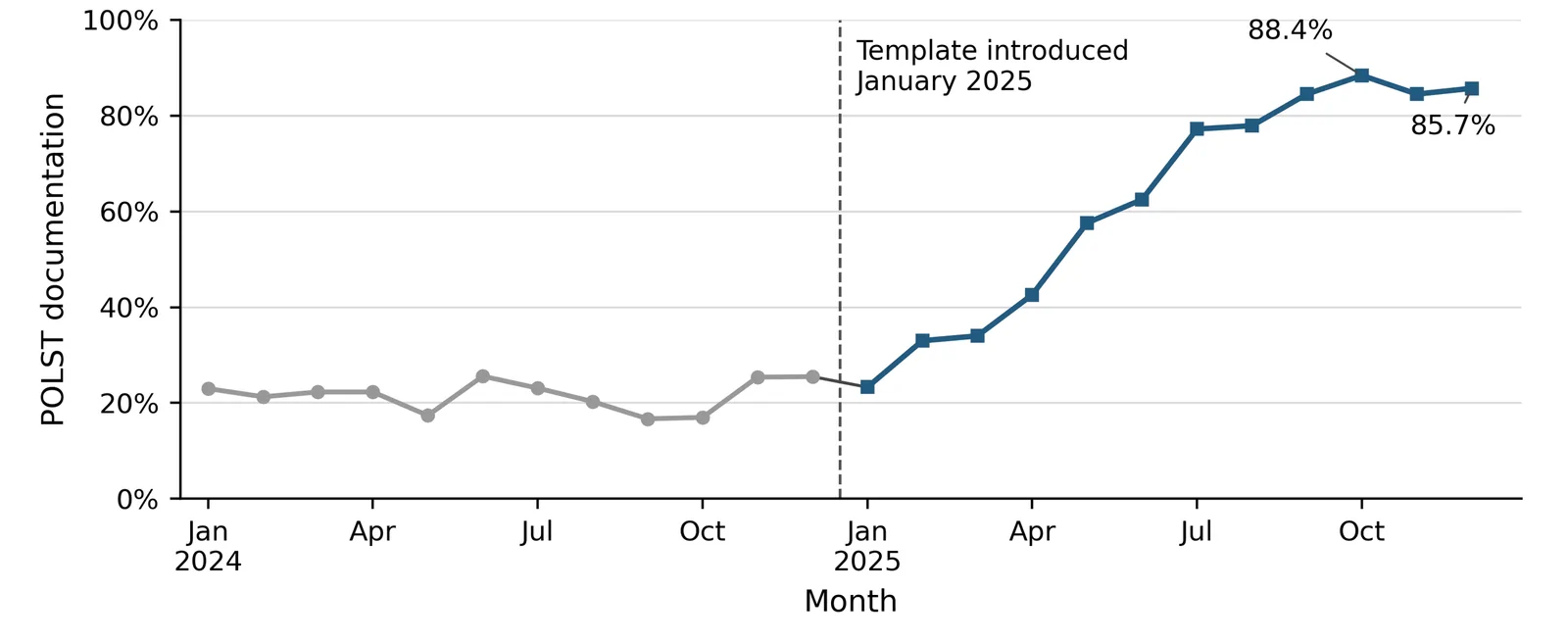
Monthly POLST documentation for Provider C Provider C monthly percentages from January 2024 through December 2025; the dashed line marks template introduction in January 2025. The monthly plot is descriptive and has no control limits. It does not establish special-cause variation. POLST: Physician Orders for Life-Sustaining Treatment

Clinician-reported workflow

Provider C described sitting with the patient and/or surrogate, explaining the POLST sections in plain language, inviting questions, and helping willing patients complete the form during the same visit when appropriate. The original form was given to the patient, and a copy was scanned into Epic Media. This account of the investigator's practice suggested candidate changes for the next improvement cycle. The contribution of these practices to documentation performance was not independently tested. The EHR template and its POLST prompts are shown in Figure 3.

**Figure 3:**
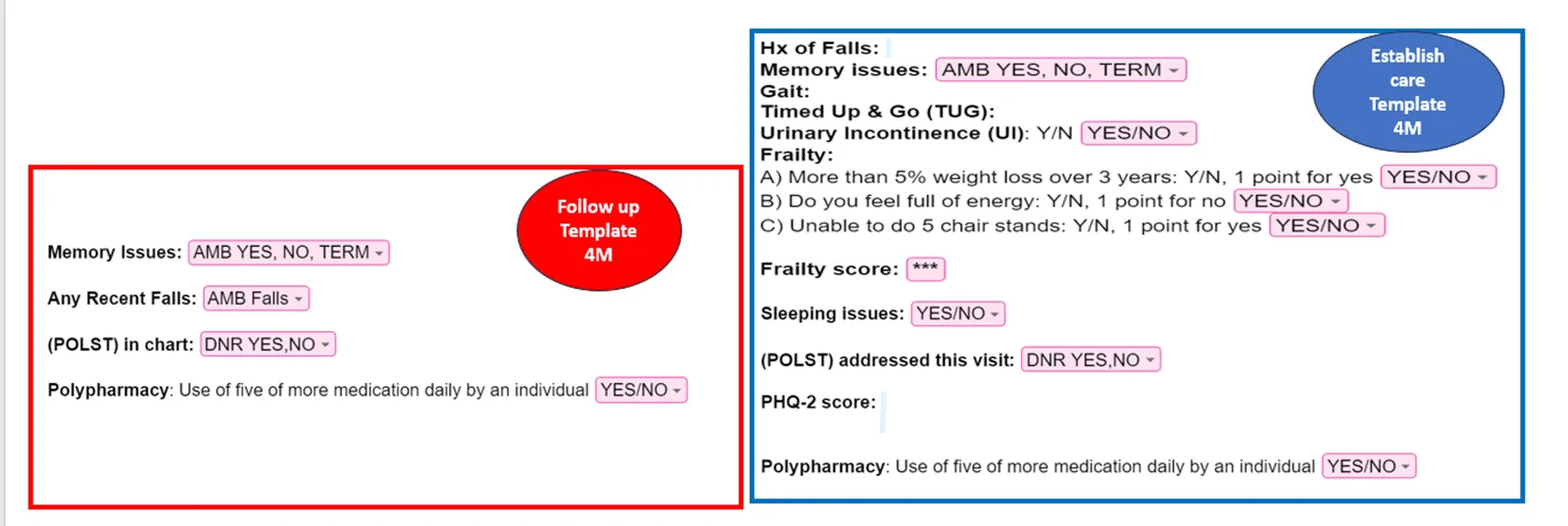
EHR template prompts The establish-care template displays the prompt "(POLST) addressed this visit". The follow-up template displays "(POLST) in chart". The "DNR YES,NO" text is the displayed label for one component of the documentation interface. Depending on the response selected, additional dropdown options were available to document POLST treatment preferences, including the level of medical intervention (full treatment, selective treatment, or comfort-focused treatment) and artificially administered nutrition. Template or dropdown selections alone did not meet the study outcome, which required a scanned POLST form in Epic Media. POLST: Physician Orders for Life-Sustaining Treatment; EHR: electronic health record

## Discussion

Integrating a POLST prompt into an Age-Friendly What Matters template, together with individual provider training, staff instructions, and quarterly one-to-one feedback on 4Ms measures, was associated with an increase in reported documentation from 11.7% to 26.9%. Improvement was observed for each clinician, but the magnitude varied substantially. This pattern supports examining how the template was used in practice and whether additional support can improve consistency. The before-and-after design cannot isolate the contribution of the template, training, review and encouragement, clinician behavior, or other changes occurring during implementation.

Our findings are consistent with the direction of results from other ACP implementation efforts. A Pacific Northwest learning collaborative reported increased ACP documentation in four of six practices [8]. A Nebraska implementation study found different degrees of improvement across two primary care sites after adding ACP coordination and rapid feedback [9]. These studies used different populations, interventions, denominators, and ACP outcomes; their estimates should not be treated as directly comparable benchmarks for POLST documentation.

Randomized evidence supports testing active conversation support. Gabbard and colleagues found ACP documentation in 42.2% of patients assigned to a nurse navigator-led pathway, compared with 3.7% receiving usual care [12]. Fried and colleagues found that motivational interviewing improved engagement in a set of ACP activities compared with usual care [13]. These interventions and endpoints differ from the process measure used here, but they provide a rationale for testing coaching and team-based assistance rather than assuming that a documentation prompt alone will reliably change practice.

The large difference in provider-specific results is an implementation finding rather than proof that one conversational technique caused improvement. Provider C's account of his own approach suggests a practical change concept: allow time to discuss each section in language the patient understands and facilitate completion during the visit when appropriate. Because Provider C was also the principal investigator, this account is subject to observer and reporting bias. Clinician experience, panel composition, visit duration, staffing, and willingness to document may also explain the variation. Provider B's fellowship training early in 2024, his greater clinical availability in 2025, and the administrative time allocated to Providers A and C provide context for the changing encounter shares. The exploratory fixed-weight calculation addresses provider weighting only.

The next Plan-Do-Study-Act cycle should test a standardized POLST workflow, conversation guidance, shared examples, and clear team responsibilities to improve the reliability of appropriate discussion and documentation. Future audits should distinguish newly completed forms from reviewed forms, assess signatures and internal consistency, and record patient decisions to defer or decline. The all-encounter denominator used here measures documentation coverage and is not an appropriateness-adjusted completion target. Completion should remain voluntary and guided by clinical appropriateness, with national guidance emphasizing serious illness or advanced frailty rather than age alone [1].

More documentation is not sufficient evidence of better end-of-life care. EHR process measures can help teams monitor implementation, but their validity as indicators of care quality requires further evaluation [6,7]. Future work should assess whether orders remain current and accessible across settings, reflect patients' informed preferences, and are honored during care transitions. Observational POLST studies show that discordant care can occur even when treatment-limiting orders are documented [2-4].

Limitations

This evaluation was conducted at one clinic with three physicians and no concurrent comparison group. Secular trends, other ACP initiatives, differences in clinician practice, and changes in the patient population could account for some of the observed improvement. The encounter denominator increased by approximately 50%, and provider encounter shares changed substantially. The fixed-weight calculation addresses only provider weighting and does not correct for patient-level differences.

The analysis used aggregate encounter data and did not estimate a unique-patient completion rate or account for repeated documentation of existing forms. Existing forms could contribute at subsequent visits, so changes in documentation may reflect accumulating form availability as well as new completion. The same IT searches and manual verification were used in both years, but the new workflow may have increased the scanning or visibility of pre-existing forms. The aggregate export did not retain encounter identifiers, document identifiers, or form timestamps, limiting independent re-audit of encounter-to-form linkage and completion dates. The measure cannot establish the rate of newly completed forms, the frequency or quality of POLST conversations, or adherence among clinically appropriate patients.

Monthly percentages were plotted for one provider only and were displayed without control limits; monthly counts for all providers are provided in Table 2. A formal clinic-wide process shift and durability beyond 2025 were not established. The workflow description was provided by the investigator whose performance improved most and was not an independent comparative assessment. The conversational practices were not tested as a separate intervention. The project did not quantify the clinical appropriateness of individual forms; an all-encounter denominator and a numerical target may encourage completion outside the intended population. The project did not measure goal-concordant treatment, healthcare utilization, patient experience, or family-reported outcomes. Balancing measures such as visit burden, pressure to complete forms, and patient understanding should be incorporated in future cycles.

## Conclusions

An Age-Friendly Health Systems 4Ms What Matters workflow was associated with a more than twofold increase in reported encounter-level POLST documentation in an outpatient geriatric clinic. Improvement varied substantially among clinicians. These findings support testing a standardized workflow and conversation support while preserving appropriate, voluntary POLST use. This uncontrolled aggregate evaluation does not establish causality, new form completion, improved conversations, or goal-concordant care. A stronger evaluation should retain encounter-level linkage and form-completion dates, account for repeated encounters and patient mix, and assess form quality and patient outcomes.
